# Urban–rural disparities in diagnosis, treatment, and prognosis of primary bone cancer: An observational study

**DOI:** 10.1097/MD.0000000000045548

**Published:** 2025-10-24

**Authors:** Chenbo Ouyang, Xuanwen Liu, Chunyu Chen, Changping Chen

**Affiliations:** a Department of Orthopedic, The Affiliated Chengdu 363 Hospital of Southwest Medical University, Chengdu, China; b Department of Neurosurgery, The Affiliated Chengdu 363 Hospital of Southwest Medical University, Chengdu, China.

**Keywords:** diagnosis, primary bone cancer, residence, survival, treatment

## Abstract

Residence may influence cancer management. However, the role of residence in primary bone cancer is not well explored. In this study, patients diagnosed with primary bone cancer were identified from the surveillance, epidemiology, and end results (SEER) database and divided into urban and rural groups based on residence. Multivariable ordinal logistic regression was used to determine the relationship between residence and stage at diagnosis. Multivariable logistic regression was used to explore the association between residence and receipt of local surgery, radiotherapy, and chemotherapy. Propensity score matching (PSM) was used to balance the baseline between the 2 groups, and Kaplan–Meier curves were used to estimate the overall survival (OS) and cancer-specific survival (CSS) of the 2 groups. A total of 13,876 patients with primary bone cancer were included. Compared with urban patients, rural patients were less likely to receive local surgery (OR = 0.78, 95% CI: 0.70–0.89, *P* < .001), radiotherapy (OR = 0.69, 95% CI: 0.60–0.88, *P* < .001), and chemotherapy (OR = 0.85, 95% CI: 0.77–0.94, *P* < .001). After PSM, rural patients had significantly worse OS (HR = 1.10, 95% CI: 1.03–1.19, *P* = .029) and CSS (HR = 1.08, 95% CI: 1.02–1.18, *P* = .036) than urban patients. However, residence was not associated with the stage at diagnosis (Rural vs Urban, OR = 1.00, 95% CI: 0.88–1.14, *P* = .989). In conclusion, rural residence is associated with lower likelihood of receiving definitive treatments (local surgery, radiotherapy, and chemotherapy) and worse survival for primary bone cancer. However, residence is not associated with stage at diagnosis.

## 1. Introduction

Primary bone cancer is a rare malignancy, accounting for approximately 0.2% of all new cancer cases.^[1,2]^ However, despite its rarity, it exhibits a distinct age-specific incidence, with higher rates observed in children and adolescents.^[3]^ Currently, the treatment of primary bone cancer primarily relies on multimodal approaches, including surgical resection, radiotherapy, chemotherapy, and targeted therapy.^[3–5]^ Despite advances in treatment in recent years, the prognosis for some types of patients remains poor due to the diverse biological behaviors of bone cancer.^[6,7]^

The diagnosis, treatment, and prognosis of cancer are influenced by various factors. In addition to the biological characteristics of the tumor, external factors such as the patient’s socioeconomic background may also have a profound impact.^[8,9]^ In recent years, the issue of healthcare inequality between urban and rural areas has garnered widespread attention. Numerous studies have shown significant disparities in the distribution of healthcare resources, with urban areas typically having more advanced medical facilities, cutting-edge treatments, and higher levels of healthcare services. In contrast, patients in rural areas often face challenges such as limited access to medical resources, difficulty obtaining care, and poorer treatment outcomes.^[10–13]^ For cancers, such urban-rural disparities may affect various aspects of cancer management.^[14,15]^ For instance, previous studies have shown that urban residents have advantages in early screening and treatment for common cancers, such as lung and breast cancer, whereas rural patients face greater diagnostic delays and fewer treatment options.^[16]^ However, this phenomenon has not been well explored in primary bone cancer.

Investigating the impact of residential location on the diagnosis, treatment, and survival outcomes of primary bone cancer patients could provide valuable insights for optimizing treatment strategies and reducing the urban–rural healthcare gap. Therefore, based on the surveillance, epidemiology, and end results (SEER) database, this study aims to explore the effects of residential location on the diagnosis, treatment, and prognosis of primary bone cancer.

## 2. Patients and methods

### 2.1. Data source and patient selection

The SEER database, established by the National Cancer Institute, is a critical public health resource designed to provide extensive data on cancer incidence, survival rates, treatments, and prognosis. Since 1973, the SEER database has covered cancer data from multiple regions across the United States and currently represents approximately half of the United States population.^[17]^ Due to its large sample size, broad coverage, and detailed data, the SEER database is particularly well-suited for studying rare malignancies. In this study, we utilized the SEER-17 register dataset, which covers approximately 26.5% of the U.S. population.^[18]^

Primary bone cancer (C40 and C41) was identified based on International Classification of Diseases for Oncology, 3rd edition codes. Given that some primary bone cancers, such as osteosarcoma, are common among children, this study included patients across all age groups. Additionally, only patients diagnosed between 2000 and 2019 were included to ensure sufficient follow-up time. Patients diagnosed solely through autopsy or death certificates, as well as those lacking key demographic data (e.g., race, marital status, median household income, or residence) or oncological outcome information, were excluded from the study. These excluded cases represent <5% of the total population. The analysis utilized de-identified, publicly accessible data from the SEER database. As a result, institutional review board approval was waived, and informed consent was exempted.

### 2.2. Variable processing

Given the age-specific nature of primary bone cancer, age was categorized into 3 groups: <15 years (childhood), 15 to 39 years (adolescents and young adults, AYA), and >39 years (adults).^[19]^ Marital status at diagnosis was classified as married and unmarried. The median household income in the SEER-17 dataset was adjusted to 2022 levels, and income was categorized based on the 2022 U.S. median household income into <75,000 USD and >75,000 USD. The histological type was determined using International Classification of Diseases for Oncology, 3rd edition histology codes. Osteosarcoma (codes 9180-9186, 9192-9194) and Ewing sarcoma (code 9260) were classified separately, considering them to be relatively common, while the other rare types were all classified as “other.”

Due to patient‐privacy protections, the SEER Public Use File does not disclose county identifiers directly; instead, it employs the U.S. Department of Agriculture Economic Research Service’s Rural–Urban Continuum Codes (RUCC) to characterize geographic attributes. The RUCC framework classifies all U.S. counties into 9 categories: 3 metropolitan groups based on metropolitan area population thresholds, and 6 nonmetropolitan groups defined by their level of urbanization and proximity to metropolitan areas. To streamline analysis and preserve statistical power, SEER aggregates these into a five‐level RUCC5 variable in the Public Use File (counties in metro areas ≥ 1 million; 250,000–1 million; <250,000; nonmetro adjacent; nonmetro nonadjacent).^[20,21]^ In our study, we defined urban residence as RUCC5 levels 1 to 3 and rural residence as levels 4 to 5, a dichotomization widely used in cancer epidemiology to reflect differences in population density, resource availability, and access to care.

### 2.3. Statistical analysis

Based on patients’ residence, the cohort was divided into 2 groups: Urban and Rural. Differences in baseline characteristics between the 2 groups were tested using Pearson χ^2^ test. Diagnostic stage was treated as an ordinal dependent variable, with categories of localized, regional, and distant. A multivariable ordinal logistic regression model was used to explore the relationship between residence and the stage at diagnosis (localized, regional, distant). Adjusted covariates included: year of diagnosis, age, sex, race, marital status, median household income, grade, and histology. Subsequently, multivariable logistic regression was used to investigate the relationship between residence and receipt of local surgery, radiotherapy, and chemotherapy. Adjusted covariates included: year of diagnosis, age, sex, race, marital status, median household income, stage, grade, and histology. Kaplan–Meier curves were then used to evaluate overall survival (OS) and cancer-specific survival (CSS). To address potential bias due to baseline imbalance, 1:1 propensity score matching (PSM) was performed. The matched variables included year of diagnosis, age, sex, race, marital status, median household income, stage, grade, histology, and treatment (local surgery, radiotherapy and chemotherapy).

Kaplan–Meier curves were again employed to assess OS and CSS between urban and rural patients after PSM. Considering the age-specific nature of primary bone cancer and the potential survival differences between urban and rural patients based on income levels, subgroup analyses by age and median household income were conducted. Additionally, subgroup analysis by year of diagnosis was performed to evaluate temporal trends in survival differences. All statistical analyses were conducted using R software (version 4.2.2), and a two-tailed *P*-value < .05 was considered statistically significant.

## 3. Results

### 3.1. Patient baseline characteristics

As shown in Table 1, a total of 13,876 patients were included in this study, with 12,430 urban patients (89.6%) and 1446 rural patients (10.4%). Before PSM, there were significant differences between rural and urban patients in several variables. Compared to urban patients, rural patients were more likely to be over 39 years old (53.5% vs 48.2%, *P* < .001), White (89.6% vs 81.2%, *P* < .001), and married (44.1% vs 39.7%, *P* = .001). Notably, there was a significant income disparity between the 2 groups. Among urban patients, 64.7% had a median household income >75,000 USD, while only 7.5% of rural patients had a median household income above this threshold. Additionally, there were significant differences between the groups in terms of year of diagnosis (*P* = .048), local surgery (*P* < .001), radiotherapy (*P* < .001), and chemotherapy (*P* = .018). After PSM, both rural and urban patients each consisted of 1446 individuals, and the groups were balanced in all baseline characteristics (all *P* > .05, all standardized mean difference^[20,21]^ <0.1).

**Table 1:** Patient baseline characteristics before and after propensity score matching.

Characteristics	Unmatched				Matched			
	Urban, N = 12,430*	Rural, N = 1446*	*P*-value†	SMD	Urban, N = 1446*	Rural, N = 1446*	*P*-value†	SMD
Year of diagnosis								
2000–2009	5805 (46.7%)	715 (49.4%)	.048	0.055	696 (48.1%)	715 (49.4%)	.480	0.026
2010–2019	6625 (53.3%)	731 (50.6%)		−0.055	750 (51.9%)	731 (50.6%)		−0.026
Age, yr								
˂15	2229 (17.9%)	236 (16.3%)	<.001	−0.044	233 (16.1%)	236 (16.3%)	.662	0.006
15–39	4205 (33.8%)	436 (30.2%)		−0.080	416 (28.8%)	436 (30.2%)		0.030
˃39	5996 (48.2%)	774 (53.5%)		0.106	797 (55.1%)	774 (53.5%)		−0.032
Sex								
Male	7011 (56.4%)	820 (56.7%)	.825	0.006	806 (55.7%)	820 (56.7%)	.600	0.020
Female	5419 (43.6%)	626 (43.3%)		−0.006	640 (44.3%)	626 (43.3%)		−0.020
Race								
White	10,091 (81.2%)	1296 (89.6%)	<.001	0.277	1304 (90.2%)	1296 (89.6%)	.708	−0.018
Black	1258 (10.1%)	98 (6.8%)		−0.133	98 (6.8%)	98 (6.8%)		0.000
Others	1081 (8.7%)	52 (3.6%)		−0.274	44 (3.0%)	52 (3.6%)		0.030
Marital status								
Married	4929 (39.7%)	637 (44.1%)	.001	0.089	649 (44.9%)	637 (44.1%)	.653	−0.017
Unmarried	7501 (60.3%)	809 (55.9%)		−0.089	797 (55.1%)	809 (55.9%)		0.017
Median household income								
˂75,000 USD	4389 (35.3%)	1337 (92.5%)	<.001	1.992	1337 (92.5%)	1337 (92.5%)	>.999	0.000
˃75,000 USD	8041 (64.7%)	109 (7.5%)		−1.992	109 (7.5%)	109 (7.5%)		0.000
Stage								
Localized	4121 (33.2%)	464 (32.1%)	.420	−0.023	480 (33.2%)	464 (32.1%)	.917	−0.024
Regional	3463 (27.9%)	388 (26.8%)		−0.023	383 (26.5%)	388 (26.8%)		0.008
Distant	1976 (15.9%)	234 (16.2%)		0.008	235 (16.3%)	234 (16.2%)		−0.002
Unknown/unstaged	2870 (23.1%)	360 (24.9%)		0.042	348 (24.1%)	360 (24.9%)		0.019
Grade								
Grade I–II	2753 (22.1%)	342 (23.7%)	.426	0.035	348 (24.1%)	342 (23.7%)	.964	−0.010
Grade III–IV	3889 (31.3%)	446 (30.8%)		−0.010	442 (30.6%)	446 (30.8%)		0.006
Unknown	5788 (46.6%)	658 (45.5%)		−0.021	656 (45.4%)	658 (45.5%)		0.003
Histology								
Osteosarcoma	4215 (33.9%)	440 (30.4%)	.024	−0.076	448 (31.0%)	440 (30.4%)	.947	−0.012
Ewing sarcoma	1628 (13.1%)	208 (14.4%)		0.037	205 (14.2%)	208 (14.4%)		0.006
Others	6587 (53.0%)	798 (55.2%)		0.044	793 (54.8%)	798 (55.2%)		0.007
Local surgery								
Yes	9671 (77.8%)	1067 (73.8%)	<.001	−0.091	1084 (75.0%)	1067 (73.8%)	.469	−0.027
No/unknown	2759 (22.2%)	379 (26.2%)		0.091	362 (25.0%)	379 (26.2%)		0.027
Radiotherapy								
Yes	3731 (30.0%)	336 (23.2%)	<.001	−0.009	307 (21.2%)	336 (23.2%)	.195	0.047
No/unknown	8699 (70.0%)	1110 (76.8%)		0.009	1139 (78.8%)	1110 (76.8%)		−0.047
Chemotherapy								
Yes	5951 (47.9%)	645 (44.6%)	.018	−0.066	638 (44.1%)	645 (44.6%)	.793	0.010
No/unknown	6479 (52.1%)	801 (55.4%)		0.066	808 (55.9%)	801 (55.4%)		−0.010

SMD = standardized mean difference, USD = United States Dollar.

* n (%).

† Pearson χ^2^ test.

### 3.2. Relationship between residence and cancer stage

Table 2 presents the results of the ordinal logistic regression analysis. In the multivariable analysis, patients diagnosed between 2010 and 2019 (2000–2009 as the reference; OR = 0.84, 95% CI: 0.78–0.91, *P* < .001), older patients (<15 as the reference; 15–39, OR = 0.75, 95% CI: 0.69–0.86, *P* < .001; >39, OR = 0.65, 95% CI: 0.58–0.71, *P* < .001), females (male as the reference; OR = 0.82, 95% CI: 0.76–0.88, *P* < .001), and patients with other histologic types (osteosarcoma as the reference; OR = 0.76, 95% CI: 0.69–0.84, *P* < .001) were more likely to be diagnosed at an earlier stage. Conversely, patients who were unmarried (married as the reference; OR = 1.16, 95% CI: 1.05–1.30, *P* < .001), had Grade III–IV cancers (Grade I–II as the reference; OR = 2.85, 95% CI: 2.50–3.13, *P* < .001), or had Ewing sarcoma (osteosarcoma as the reference; OR = 1.65, 95% CI: 1.47–1.86, *P* < .001) were more likely to be diagnosed at a later stage. However, residence (rural vs urban, OR = 1.00, 95% CI: 0.88–1.14, *P* = .989) was not identified as an independent prognostic factor for cancer stage.

**Table 2:** Ordered logistic regression identifies factors affecting staging of patients with primary bone cancers.

Characteristic	Univariable		Multivariable	
	OR (95% CI)	*P*-value	OR (95% CI)	*P*-value
Year of diagnosis				
2000–2009	Reference	<.001	Reference	<.001
2010–2019	0.86 (0.80–0.92)		0.84 (0.78–0.91)	
Age				
˂15	Reference		Reference	
15–39	0.78 (0.71–0.87)	<.001	0.75 (0.69–0.86)	<.001
˃39	0.69 (0.62–0.76)	<.001	0.65 (0.58–0.71)	<.001
Sex				
Male	Reference	<.001	Reference	<.001
Female	0.80 (0.74–0.86)		0.82 (0.76–0.88)	
Race				
White	Reference		Reference	
Black	1.00 (0.89–1.13)	.951	1.00 (0.88–1.13)	.976
Others	1.07 (0.94–1.22)	.276	1.04 (0.91–1.18)	.592
Marital status				
Married	Reference	<.001	Reference	<.001
Unmarried	1.27 (1.18–1.36)		1.16 (1.05–1.30)	
Median household income				
˂70,000 USD	Reference		Reference	
70,000–85,000 USD	0.99 (0.91–1.08)	.873	0.98 (0.89–1.07)	.611
˃85,000 USD	0.96 (0.88–1.05)	.422	0.95 (0.86–1.05)	.339
Residence				
Urban	Reference	.640	Reference	.989
Rural	1.03 (0.91–1.16)		1.00 (0.88–1.14)	
Grade				
Grade I–II	Reference		Reference	
Grade III–IV	3.24 (2.92–3.59)	<.001	2.80 (2.50–3.13)	<.001
Unknown	2.32 (2.11–2.55)	<.001	1.98 (1.79–2.19)	<.001
Histology				
Osteosarcoma	Reference		Reference	
Ewing sarcoma	1.65 (1.47–1.86)	<.001	1.85 (1.64–2.10)	<.001
Others	0.60 (0.56–0.65)	<.001	0.76 (0.69–0.84)	<.001

USD = United States Dollar.

### 3.3. Relationship between residence and treatment

As shown in Table 3, in the univariate logistic regression analysis, rural patients were less likely to receive local surgery (OR = 0.80, 95% CI: 0.71–0.91, *P* < .001), radiotherapy (OR = 0.71, 95% CI: 0.63–0.81, *P* < .001), and chemotherapy (OR = 0.88, 95% CI: 0.79–0.98, *P* = .014) compared to urban patients. After adjusting for covariates, rural patients remained less likely to receive local surgery (OR = 0.78, 95% CI: 0.70–0.89, *P* < .001), radiotherapy (OR = 0.69, 95% CI: 0.60–0.88, *P* < .001), and chemotherapy (OR = 0.85, 95% CI: 0.77–0.94, *P* < .001) compared to urban patients.

**Table 3:** Odds ratios for receiving specific treatments in patients with primary bone cancers.

Treatment	Univariate*		Multivariate†	
	OR (95% CI)	*P*-value	OR (95% CI)	*P*-value
Local surgery	0.80 (0.71–0.91)	<.001	0.78 (0.70–0.89)	<.001
Radiotherapy	0.71 (0.63–0.81)	<.001	0.69 (0.60–0.88)	<.001
Chemotherapy	0.88 (0.79–0.98)	.018	0.85 (0.77–0.94)	<.001

* Odds ratios for receiving specific treatments (local surgery, radiotherapy, chemotherapy) in patients living in rural areas compared with patients living in urban areas.

† Adjusted covariates included year of diagnosis, age, sex, race, marital status, median household income, residence, stage, grade, and histology.

### 3.4. Relationship between residence and survival

**Figure 1.**
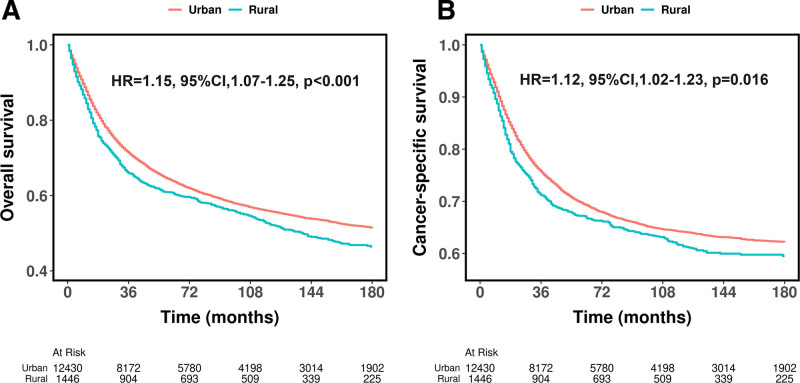
Kaplan–Meier curves for overall survival (A) cancer-specific survival (B) of patients diagnosed with primary bone cancer before propensity score matching.

**Figure 2.**
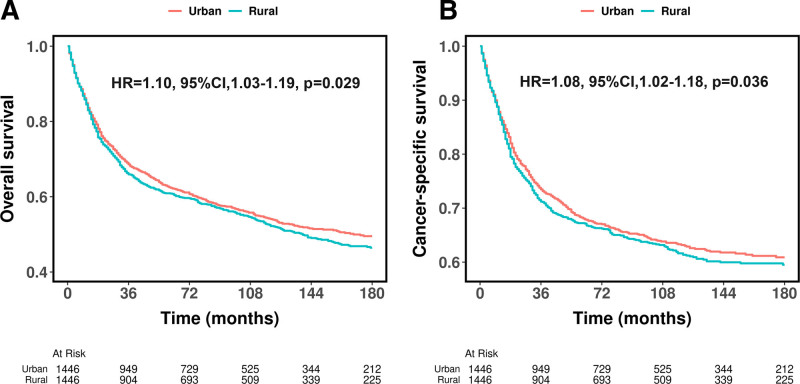
Kaplan–Meier curves for overall survival (A) cancer-specific survival (B) of patients diagnosed with primary bone cancer after propensity score matching.

As shown in Figure 1, rural patients exhibited worse OS (HR = 1.15, 95% CI: 1.07–1.25, *P* < .001, Fig. 1A) and CSS (HR = 1.12, 95% CI: 1.05–1.23, *P* = .016, Fig. 1B) compared to urban patients before PSM. After PSM, rural patients still demonstrated significantly worse OS (HR = 1.10, 95% CI: 1.03–1.19, *P* = .029, Fig. 2A) and CSS (HR = 1.08, 95% CI: 1.02–1.18, *P* = .036, Fig. 2B) compared to urban patients, though the differences were slightly attenuated. Table S1 (Supplemental Digital Content, https://links.lww.com/MD/Q471) presents the OS and CSS rates at 36, 60, 120, and 180 months before and after PSM, with rural patients consistently showing worse survival at all time points.

**Figure 3.**
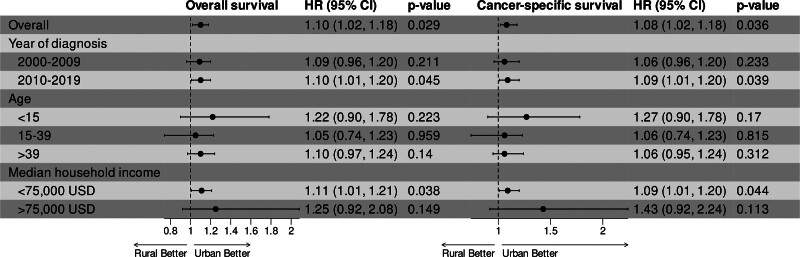
Subgroup analysis based on diagnosis year, age, and income level of overall survival and cancer-specific survival in patients diagnosed with primary bone cancer after propensity score matching.

Figure 3 shows the results of subgroup analysis based on key variables (diagnosis year, age, and median household income). While a trend of worse survival for rural patients was observed in all subgroups (all HR > 1), significant differences were only observed in patients diagnosed between 2010–2019 (OS: HR = 1.10, 95% CI: 1.01–1.20, *P* = .045; CSS: HR = 1.09, 95% CI: 1.01–1.20, *P* = .039) and those with a median household income <75,000 USD (OS: HR = 1.11, 95% CI: 1.01–1.21, *P* = .038; CSS: HR = 1.09, 95% CI: 1.01–1.20, *P* = .044).

## 4. Discussion

In primary bone cancer, in addition to biological factors, previous studies have shown that several socioeconomic factors also affect patient prognosis.^[22–24]^ For example, Huang et al and Wen et al found that married patients had better survival than unmarried patients with primary bone cancer.^[22,23]^ Shi et al found that female had better CSS (HR = 0.86, 95% CI: 0.79–0.94, *P* < .001) and OS (HR = 0.83, 95% CI: 0.77–0.89, *P* < .001) compared with male with primary bone cancer.^[24]^ Potential reasons for these phenomena are multifactorial, including greater family support and greater treatment compliance. But ultimately, these improvements in survival are mainly due to the diagnostic and treatment advantages of access to medical services.^[22–24]^

As an important socioeconomic factor, the role of residence in the diagnosis, treatment, and prognosis of various malignancies has been well explored.^[16]^ For cancer diagnosis, Zahnd et al using the North American Association of Central Cancer Registries public use data set found that, for all cancers, rural populations had a lower incidence of localized-stage cancer compared to urban populations (OR = 0.95, 95% CI: 0.95–0.95), while the incidence of distant-stage cancer was higher (OR = 1.05, 95% CI: 1.05–1.06).^[25]^ In a large Chinese cohort study of lung cancer, gastric cancer, esophageal cancer, colorectal cancer, and female breast cancer, rural patients had a higher proportion of advanced disease than urban patients (OR = 1.2, 95% CI: 1.1–1.4).^[14]^ In terms of treatment, rural patients are less likely to receive curative treatments compared to their urban counterparts.^[16,26,27]^ Based on the National Cancer Database, Logan et al found that among patients with resectable non-small cell lung cancer, rural patients were more likely to not undergo surgery than urban patients (OR = 1.21, 95% CI: 1.13–1.29, *P* < .001).^[26]^ In terms of oncological outcomes, rural residence has been found to be associated with worse survival in multiple cancers.^[15,28–30]^

The disparities in cancer outcomes between urban and rural populations may stem from multiple interrelated factors, such as unequal distribution of medical resources, differences in patients’ health literacy, and barriers to health care such as transportation.

Among these, the uneven distribution of oncology resources is a key contributor. Previous studies have shown that oncologists and surgical specialists are disproportionately concentrated in metropolitan areas, with rural regions having only about half the oncologist density compared to urban areas.^[15]^ In addition, there is a severe lack of rare cancer treatment centers and clinical trial opportunities in rural areas, resulting in rural patients being 20 to 30% less likely to participate in clinical trials than urban patients.^[31]^ Due to limited availability of local treatment options, rural patients often have to travel long distances to access care. For example, a SEER-Medicare-based study found that elderly breast cancer patients residing in rural areas had nearly 3 times the average travel distance compared to urban patients (40.8 miles vs 15.4 miles), and the nearest healthcare facility was more than 4 times farther away (21.9 miles vs 4.8 miles).^[32]^ Additionally, over 7% of rural Medicare beneficiaries cross state lines to access specialized cancer care, a rate nearly double that of urban patients.^[33]^ Health literacy also differs substantially between rural and urban populations. Rural cancer patients are 33% more likely to have low health literacy, which may negatively impact their understanding of complex treatment regimens and adherence.^[34]^ Lindert et al reported that rural workers, due to the nature of physical labor and rigid work schedules, had significantly lower participation rates in health promotion programs compared to urban workers.^[35]^ Agricultural laborers, in particular, often attribute health symptoms to physical fatigue and delay seeking professional medical care due to long working hours and financial constraints.^[36]^ Transportation remains a major barrier to healthcare access in rural communities. A previous report estimated that 5.8 million Americans delayed or forgo medical care due to lack of transportation, with rural residents disproportionately affected.^[37]^ Rural cancer patients often travel over an hour one-way to reach treatment facilities, leading to higher rates of missed appointments and treatment delays.^[38]^ For instance, in Pennsylvania, the average round-trip travel time for patients in RUCC 1-3 areas was 41.5 minutes, compared to 128.9 minutes for those in RUCC 7-9 areas.^[39]^ Collectively, these factors contribute to the significant urban-rural disparities in cancer management.

Previous studies have explored urban-rural disparities in the diagnosis and prognosis of primary bone cancers such as Ewing sarcoma and osteosarcoma.^[40–42]^ For instance, Alsoof et al utilized the SEER database to analyze Ewing sarcoma patients between 2005 and 2019, finding that those residing in non-metropolitan areas presented with larger tumors and had a higher cancer-specific mortality risk compared to metropolitan residents.^[40]^ Similarly, McMahon et al, using data from the National Cancer Database (NCDB), reported that patients with Ewing sarcoma living in rural areas exhibited increased mortality within the first 2 years post-diagnosis.^[41]^ Blakey et al reported that patients with osteosarcoma and Ewing sarcoma who lived in more remote rural areas in the United Kingdom had a higher risk of early death.^[42]^ However, these studies often had limitations, such as relatively small sample sizes or a focus on specific subtypes of bone cancer, and many lacked comprehensive analysis encompassing diagnosis, treatment, and prognosis.

In this study, we leveraged the extensive SEER database to conduct a comprehensive analysis of the association between residential location and the stage at diagnosis of primary bone cancer. We found that rural patients were less likely to receive definitive treatment (such as local surgery, radiotherapy, and chemotherapy) compared with urban patients, and were associated with worse survival (including CSS and OS). These results suggest that for patients with primary bone cancer, especially those in rural areas, improving access to treatment after diagnosis may be a key measure to improve the survival rate of rural patients and narrow the urban–rural survival gap. In the subgroup analysis based on diagnosis year, age, and median household income, significant urban-rural survival differences were observed only among patients diagnosed between 2010 and 2019, and those with a median household income below 75,000 USD. However, it is worth noting that a trend toward urban-rural survival differences was observed in all subgroups (HR > 1), although this trend did not reach statistical significance in most subgroups. Considering the relatively small sample size of the subgroup analysis, this may be due to insufficient statistical power caused by sample size limitations. Interestingly, we found that residential location was not significantly associated with cancer stage at diagnosis in patients with primary bone cancer. One possible explanation is that bone cancers often present with overt symptoms such as pain or functional impairment, prompting patients to seek medical attention relatively quickly regardless of where they live. As a result, both urban and rural patients may be less likely to experience delays that would lead to more advanced-stage diagnoses, thereby minimizing differences in stage distribution. Moreover, the SEER program’s sampling and case registration methods tend to cover counties with at least basic diagnostic infrastructure, which may limit its ability to capture cases from the most remote or resource-deprived areas. This limitation could statistically obscure subtle urban-rural differences in staging data.^[15]^ Together, these factors may help explain the lack of a significant disparity in stage at diagnosis between rural and urban patients observed in our study.

In conclusion, our study highlights disparities in outcomes between urban and rural patients with primary bone cancer and underscores the need for targeted efforts to reduce these gaps. First, given the existing disparities in healthcare access between rural and urban areas, telemedicine represents a promising solution. Through virtual consultations and remote counseling, rural patients can receive expert guidance from oncology specialists at urban cancer centers without leaving their communities. Previous studies have shown that digital health technologies significantly improve access to and continuity of cancer care in rural settings.^[43]^ Second, implementing patient navigation programs can offer rural patients coordinated, one-stop support for scheduling, transportation, and follow-up, thereby enhancing healthcare accessibility and optimizing treatment outcomes, particularly for socioeconomically disadvantaged cancer patients.^[44]^ Third, expanding transportation support through community transit partnerships or travel subsidies can help address missed or delayed care due to a lack of transportation. Finally, establishing regional care networks, including satellite clinical trial sites and mobile outreach by oncology specialists, may increase rural participation in clinical research and broaden access to high-quality treatment.^[45]^ Collectively, these strategies, deployed at the system, community, and individual levels, hold promises for meaningfully improving treatment accessibility and survival outcomes among rural patients with primary bone cancer.

## 5. Limitations

Several limitations should be acknowledged. First, although the SEER-17 database covers approximately 26.5% of the U.S. population, it may underrepresent the most remote or underserved rural areas, which could lead to an underestimation of the true extent of rural–urban disparities. Second, SEER lacks detailed socioeconomic data such as insurance status, income level, education, and employment, all of which are closely related to healthcare access and treatment adherence. Third, the database does not contain information on treatment facility locations or patient referral patterns, preventing us from assessing whether rural patients traveled to urban centers for care or from analyzing the regional distribution of healthcare resources. Moreover, SEER does not capture other important structural barriers such as transportation difficulties, clinical trial accessibility, or health literacy differences, all of which may contribute to disparities in cancer outcomes. Lastly, although we applied PSM to reduce baseline differences between rural and urban groups, unmeasured confounders may still exist, leading to potential residual bias. Despite these limitations, our study provides valuable population-based evidence and highlights the need for more granular data to better understand and address rural-urban disparities in cancer care.

## 6. Conclusion

Among patients with primary bone cancer, rural patients were less likely to receive definitive treatment (local surgery, radiotherapy, and chemotherapy) and had worse survival than urban patients. However, there was no difference in stage at diagnosis between rural and urban patients. More studies are needed to further validate our findings and explore potential factors that influence treatment decisions in rural patients.

## Acknowledgments

We would like to express our gratitude to the SEER program, which provided the comprehensive data used in this study.

## Author contributions

**Conceptualization:** Chenbo Ouyang, Xuanwen Liu, Chunyu Chen, Changping Chen.

**Data curation:** Chenbo Ouyang.

**Formal analysis:** Chenbo Ouyang.

**Methodology:** Chenbo Ouyang.

**Supervision:** Changping Chen.

**Writing – original draft:** Chenbo Ouyang, Xuanwen Liu, Chunyu Chen, Changping Chen.

**Writing – review & editing:** Chenbo Ouyang, Changping Chen.

## Correction

This article has been published with an incorrect academic degree of all authors in the author group. The academic degree has been now updated online from “MD” to “MMed” for authors Chenbo Ouyang, Xuanwen Liu, Chunyu Chen, Changping Chen.

## Supplementary Material


