# Supplementary material for: Urban–rural disparities in diagnosis, treatment, and prognosis of primary bone cancer: An observational study
Source: Medicine (Baltimore). 2025 Oct 24;104(43):e45548. doi: 10.1097/MD.0000000000045548 (PMC12558280; doi:10.1097/MD.0000000000045548)
Supplement: Supplementary file 1 [file medi-104-e45548-s001.docx]

**Table S1. Cancer-specific and overall survival rates by residence in primary bone cancer patients before and after propensity score matching**

| Survival Type | PSM Status | Residence | 36-months  (95% CI, %) | 60-months  (95% CI, %) | 120-months  (95% CI, %) | 180-months  (95% CI, %) | p-value* |
| --- | --- | --- | --- | --- | --- | --- | --- |
| OS | Before | Urban | 71.4  (70.6-72.2) | 64.4  (63.5-65.2) | 55.8  (54.9-56.8) | 51.4  (50.4-52.4) | <0.001 |
| OS | Before | Rural | 65.9  (63.5-68.4) | 60.9  (58.4-63.5) | 52.2  (49.5-55.0) | 46.4  (43.5-49.5) |  |
| CSS | Before | Urban | 75.7  (74.9-76.5) | 69.7  (68.9-70.6) | 64.2  (63.2-65.1) | 62.2  (61.2-63.2) | 0.016 |
| CSS | Before | Rural | 71.2  (68.8-73.6) | 67.2  (64.8-69.8) | 61.3  (58.7-64.2) | 59.5  (56.7-62.5) |  |
| OS | After | Urban | 69.6  (67.2-72.1) | 63.5  (61.0-66.1) | 55.1  (52.4-57.9) | 50.5  (47.6-53.6) | 0.029 |
| OS | After | Rural | 65.9  (63.5-68.4) | 60.9  (58.4-63.5) | 52.2  (49.5-55.0) | 46.4  (43.5-49.5) |  |
| CSS | After | Urban | 74.5  (72.2-76.8) | 69.3  (66.8-71.8) | 64.2  (61.5-66.9) | 61.9  (59.1-64.8) | 0.036 |
| CSS | After | Rural | 71.2  (68.8-73.6) | 67.2  (64.8-69.8) | 61.3  (58.7-64.2) | 59.5  (56.7-62.5) |  |

Abbreviation: PSM, propensity score matching; OS, overall survival; CSS, cancer-specific survival.

**^*^**Log-rank test
